# Automatic image analysis for gene expression patterns of fly embryos

**DOI:** 10.1186/1471-2121-8-S1-S7

**Published:** 2007-07-10

**Authors:** Hanchuan Peng, Fuhui Long, Jie Zhou, Garmay Leung, Michael B Eisen, Eugene W Myers

**Affiliations:** 1 Janelia Farm Research Campus, Howard Hughes Medical Institute, Ashburn, VA 20147, USA; 2 Department of Computer Science, Northern Illinois University, DeKalb, IL 60115, USA; 3 Department of Molecular and Cell Biology, University of California, Berkeley, CA 94720, USA; 4 Genomics Division, Lawrence Berkeley National Laboratory, Berkeley, CA 94720, USA

## Abstract

**Background:**

Staining the *m*RNA of a gene via *in situ *hybridization (ISH) during the development of a *D. melanogaster *embryo delivers the detailed spatio-temporal pattern of expression of the gene. Many biological problems such as the detection of co-expressed genes, co-regulated genes, and transcription factor binding motifs rely heavily on the analyses of these image patterns. The increasing availability of ISH image data motivates the development of automated computational approaches to the analysis of gene expression patterns.

**Results:**

We have developed algorithms and associated software that extracts a feature representation of a gene expression pattern from an ISH image, that clusters genes sharing the same spatio-temporal pattern of expression, that suggests transcription factor binding (TFB) site motifs for genes that appear to be co-regulated (based on the clustering), and that automatically identifies the anatomical regions that express a gene given a training set of annotations. In fact, we developed three different feature representations, based on Gaussian Mixture Models (GMM), Principal Component Analysis (PCA), and wavelet functions, each having different merits with respect to the tasks above. For clustering image patterns, we developed a minimum spanning tree method (MSTCUT), and for proposing TFB sites we used standard motif finders on clustered/co-expressed genes with the added twist of requiring conservation across the genomes of 8 related fly species. Lastly, we trained a suite of binary-classifiers, one for each anatomical annotation term in a controlled vocabulary or ontology that operate on the wavelet feature representation. We report the results of applying these methods to the Berkeley Drosophila Genome Project (BDGP) gene expression database.

**Conclusion:**

Our automatic image analysis methods recapitulate known co-regulated genes and give correct developmental-stage classifications with 99+% accuracy, despite variations in morphology, orientation, and focal plane suggesting that these techniques form a set of useful tools for the large-scale computational analysis of fly embryonic gene expression patterns.

## Background

A large body of work analyzing DNA micro-array data from microorganisms has demonstrated the value of gene expression analysis in understanding gene function and dissecting gene regulation [1-3]. While these micro-array analyses can be extended to multi-cellular organisms by serially analyzing different cell-types and tissues, such work misses the complexity of expression patterns and relationship between patterns. RNA *in situ *hybridization (ISH) provides a powerful way to visualize gene-expression patterns directly. This technique localizes specific *m*RNA sequences in tissues/cells by hybridizing a labeled complimentary nucleotide probe to the sequence of interest in fixed tissues. Visualizing the probe by colorimetric or fluorescent microscopy allows for the production of high quality images recording the spatial location and intensity of gene expression.

Traditionally such ISH images have been analyzed by direct inspection of microscope images. Several *in situ *databases, such as the Berkeley Drosophila Genome Project (BDGP) gene expression pattern database [4], record biologists' descriptions of expression patterns using controlled vocabularies [5]. With the growing size and complexity of these databases, it is desirable to complement this manual process with methods that can automatically analyze *in situ *images. Automatic analyses would make the process more rapid and consistent, and may identify biologically significant features missed during manual curation.

We focus on the automatic analysis of images of *in situ *gene expression patterns within fruit fly (*Drosophila melanogaster*) embryos. This is already a challenging task for some existing image databases. For example, the BDGP group examined the expression patterns of 5,270 genes, and recorded in their expression pattern database 56,644 images of the 3,012 genes that exhibited patterned expression at some stage of development. The problem is complicated by the fact that the morphology varies between embryos even if they are at exactly the same time point in their development. Moreover, the spatial orientation of the embryo and the particular focal plane within the 3D embryo are at the whim of the technician capturing the images. In general, there are several key analyses that are of interest:

• How to formulate and compute "features" with which to describe expression patterns that best enable the following studies:

• How to identify clusters of genes with similar spatio-temporal expression patterns?

• How to determine which genes in a cluster of co-expressed genes are co-regulated and if so what TFB sites do they have in common?

• How to annotate each gene expression pattern with respect to an anatomical ontology?

Addressing these issues provides several ways to study the expression and functions of genes based on *in situ *embryonic fly images. Segmentation and comparison of gene expression patterns assist one in understanding the activity of the enhancer regions of genes and in building models of the transcriptional control of genes based on the relationships between gradients of the expression patterns [6,7]. Further, as genes in the same pathway likely have co-localized expression, grouping genes in the image domain based on similar expression patterns, or in the domain of a controlled anatomical ontology, allows one to efficiently screen gene functions as well as detect potential regulatory elements at the sequence level.

We have developed several image analysis techniques to tackle these problems [8-10]. In these studies, to capture both the local and global properties of fly embryonic patterns in different applications, we proposed and developed three types of features: (1) Gaussian-mixture-model (GMM) "blobs", (2) the principal component eigenvectors over all images, and (3) a selected subset of the most informative [11] basis functions in a discrete, Haar-wavelet decomposition of the images. The GMM-blobs capture local properties and were used to segment the meaningful portion of each gene expression pattern. The eigenvector features capture global characteristics and are useful in identifying tight clusters of co-expressed genes. The selected wavelet features capture both global and local phenomenon and are effective as inputs to classifiers that report staging information and anatomical descriptions of the regions that are stained. With a new suite of results, this paper summarizes our computational approaches for fly gene expression pattern comparison, clustering, and classification, and the respective biological applications of automatic retrieving similar patterns, detecting gene sequence motifs, and annotation of *in situ *gene expression patterns.

There are several other recent pieces of work on comparing and clustering gene expression patterns of developing flies. For example, for the early stages (1–5) of fly embryos Kumar *et al. *binarized the image patterns and then built a retrieval system that given an image finds other similar images based on the correlation of the pixels [12], and later based on invariant moment features of the binarized images [13]. Pan *et al. *used independent component analysis to extract fly embryo features and applied it to image mining [14]. Reinitz *et al. *built a series of simplified spatio-temporal models of expression along the anterior-posterior axis for comparing and inferring the underlying regulatory mechanisms giving rise to the patterns at the cellular level [15,7,6]. Ahammad *et al. *have developed a joint-parametric alignment method for registering fly imaginal discs [16].

## Results

### Feature extraction and selection for gene expression patterns

**Figure 1:**
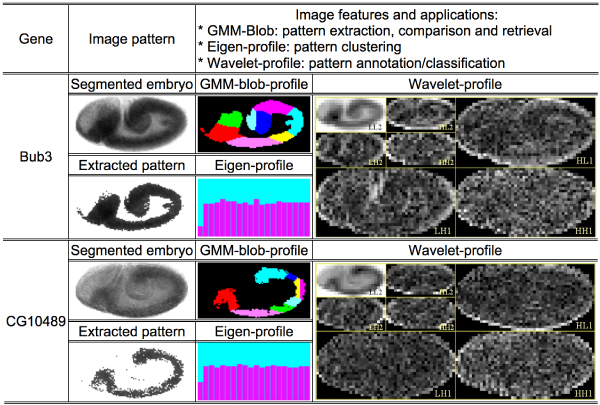
**Features extracted for the *in situ *expression patterns of two fly genes at embryonic developmental stage 7–8**. The embryos were segmented from the background using a series of image processing operations (see Data). The gene expression patterns were extracted using a global Gaussian mixture-model [8]. The GMM-blobs were then generated using the 2D local GMM [8]. Different colors in a blob-set indicate different spatial blobs. The eigen-profiles were produced using the eigen-feature decomposition [9]. The wavelet-profiles were produced using the level-2 2D Haar wavelet decomposition [10].

Figure 1 shows the three types of features we used, (1) Gaussian mixture model (GMM) blobs, (2) the eigenvector basis of the space of all images, and (3) a discrete Haar-wavelet basis. Going forward we will refer to these more briefly as GMM-blobs, eigen-features, and wavelet-features. Given a set of features, each image is described as a weighted combination of the underlying features, and the vector of weights is then considered to be the description of the image pattern with respect to the underlying feature space or basis. We call these descriptions *profiles *and will speak of blob-profiles, eigen-profiles, and wavelet-profiles.

GMM-blobs [8] are local features that combine the intensity and spatial location information of an embryo gene expression pattern adaptively. In this framework, a pattern is decomposed into a set of GMM blobs, each of which is a 2D Gaussian over a region of homogeneous intensity. Two methods can be used to produce the set of GMM-blobs for a given image. The method proposed in our original paper [8] first partitions the histogram of an image using a global 1D GMM and each partition defines a region of homogeneous intensity of the *in situ *stain. This set of regions is regarded as the gene expression pattern for this image and it is then further partitioned using a 2D GMM decomposition to obtain the set of local GMM blobs. The GMM decompositions at both steps were found using the Expectation Maximization (EM) method [17]. An alternative way to integrate the intensity and spatial information simultaneously is to treat the pixel-wise density of the *in situ *stain as being proportional to the number of photons at each pixel, so that pixel intensity can be used as the weighting coefficients in the spatial decomposition [18]. GMM-blobs provide a flexible and adaptive local representation of the gene expression patterns. Two images can be compared by matching the most similar blobs in their GMM-blob decompositions [8]. Because we used EM to estimate both the optimal parameters of Gaussian blobs and the number of Gaussians, empirically we found this approach is not sensitive to the initialization. However, GMM-blobs do not offer a canonical feature space wherein one can take advantage of the existence of the distribution of the features across all images.

Eigen-features [9] provide a global representation of embryo gene expression pattern by decomposing each pattern into a weighted combination of a *globally *selected basis vectors that are mutually orthogonal to each other. Consider the matrix whose columns are the images each linearized into a vector of pixel values. Principal Component Analysis (PCA) selects the *L *eigenvectors of this matrix corresponding to the *L *largest eigenvalues as the desired basis. Thus an image eigen-profile can be viewed as a data point in the *L*-dimensional space defined by these basis vectors, namely, the *L*-tuple of weights in the eigenvector decomposition for the image. The *L *largest eigenvectors provide a canonical subspace in which the distances between all data points can be measured. The largest eigenvectors capture the major variance in the image data, with the small variations that are ignored often corresponding to noise. It also provides a canonical space that minimizes the least-square-error incurred by removing the residues of the projection to this space from the higher-dimensional space of all eigenvectors. This eigen-feature approach was first used in human-face recognition [19], and was first used for embryo expression pattern analysis by us [9]. Unlike the wavelet-feature approach about to be discussed, the eigen-feature approach does not allow one to consider additional class/annotation information that might be associated with the images. Moreover, while there are obvious methodological niceties associated with having a fixed, global basis for all images, important local correlations may be missed.

Wavelet-features [10] characterize both the local and global information of an embryo gene expression pattern. We used a two-dimensional Discrete Wavelet Transform (DWT), which decomposes an image into an orthonormal basis of wavelet functions that are independent of the set of images. The important feature of this wavelet basis is that individual wavelets are spatially local, and cover all scales and frequencies, thus providing a local representation like the GMM-blobs with the advantage of a canonical decomposition. The one difficulty is that there are so many wavelets in the basis that the dimensionality of the DWT coefficient vector for a given image is typically huge. We reduced the dimensionality by selecting a subset of the wavelets in the basis that best help us to discriminate among the image patterns with respect to a given classification goal.

Feature selection, in general, is to select a subset of features that best discriminate between classes of image patterns. For the automatic annotation of gene expression patterns, we selected a compact wavelet feature set for a specific gene ontology annotation using the Minimum-Redundancy-Maximum-Relevance (MRMR) selection method [11]. The MRMR algorithm selects features so that their statistical dependency on the distribution of the annotations of all samples is maximized. Based on information theory, the method searches for features that are mutually far away from each other (minimum redundancy) but also maximally similar to the distribution of the annotation (maximum relevance). We used mutual information to measure the level of similarity between features. Typically, a small number of features (e.g. 20) are sufficient to well characterize the images with respect to a given annotation.

### Clusters of co-expressed genes and detection of regulatory sequence motifs

Genes that have similar functions or work together in a common pathway are likely to be under common regulatory control and thus share similar gene expression profiles or patterns. Therefore, clusters of genes that are spatially co-localized, e.g. have the same spatial pattern of expression, are more likely to be under coordinated transcriptional control, especially if the patterns unfold in the same way through time. Using such clusters of co-localized genes we can detect sequence motifs in enhancer regions that are putative TFB sites or other regulatory signals. Drosophila embryogenesis has 16 stages, which are divided into 6 major ranges, i.e. stages 1–3, 4–6, 7–8, 9–10, 11–12, and 13–16, in the BDGP database. Co-expressed genes are those sharing similar spatial-temporal expression patterns over a range of these developmental stages. We detected co-expressed genes by first clustering the image patterns within each stage-range and then identifying sets of genes that are common to the range clusters through an interval of ranges.

There are many data clustering approaches [20], such as K-Means, agglomerative hierarchical clustering [1], and graph-partition based spectral clustering [21]. For our domain, we found that graph-partitioning generated the most meaningful clusters and we developed a new graph-partition method, MSTCUT [9], to generate image clusters based on both GMM-blobs and eigen-features. We started with a weighted, undirected graph *G *= (*V*, *E*) where each node *v *∈ *V *represents an image pattern and there is an edge between each pair of nodes weighted with the Euclidean distance between the two image patterns in either the GMM-blob or eigen feature-space. The problem is to partition the graph into disjoint subgraphs each of which represents a cluster. We constrained the algorithm to partition the graph so that the resulting *K *parts are mutually distal from each other, but within each of them the average distance is as small as possible. To solve this combinatorial problem, called Min-Max Partition (MMP), efficiently, we used an approximation approach. As all edge weights are Euclidean, the triangle inequality allows us to eliminate edges with the largest distances while preserving the key cluster information in the original graph *G*. Taking this process to its logical end-point gives a minimum distance spanning tree (MST) that can be computed directly in O(|*V|*log|*V|*) time. We then produce a *K *partitioning of the tree by greedily performing the best *K*-1 bi-partitionings of the tree in O(|*V*|K) time. We have found this algorithm, which we call MSTCUT, to be quantitatively better than several other schemes for this domain [9].

**Figure 2:**
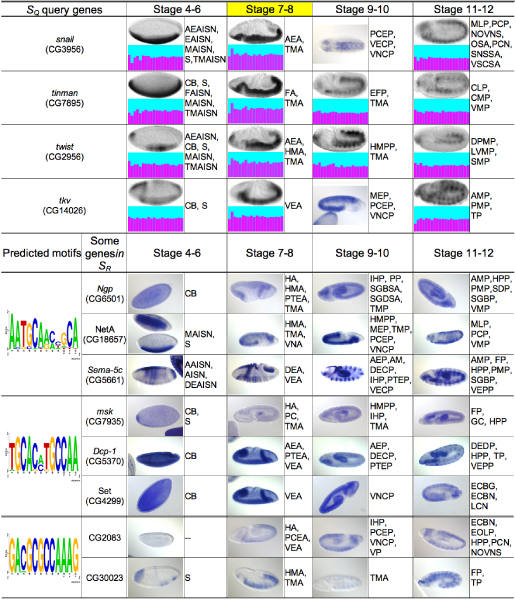
**A group of genes found by our method and examples of detected motifs along with some retrieved genes**. The gene group *S*_*Q *_was obtained by finding a tight image-cluster for stage 7–8 (highlighted) and their known biological connection is described in the text. For each gene in *S*_*Q*_, one representative embryo image is shown for each stage-range, followed by the respective eigen-profile (except for *snail *and *tkv *at stage 9–10 for which there is no appropriate lateral-view image in our data). Three motifs detected using the entire upstream regions of the homologous genes in eight fly species are shown, along with two or three randomly selected example genes in the subsequent genome-wide motif scanning results. BDGP ISH images (in blue) and abbreviations (see Appendix A) of their anatomical annotations are also shown, without image cropping or orientation correction.

For a set of 456 fly genes (see Data), we generated 90 small gene clusters based on image clustering at different developmental stage ranges, by using all the available patterns of these genes and also combining the eigen-profile clustering results simultaneously done based on both the raw images and the extracted gene expression patterns based on GMM. Figure 2 shows an example. For stage 7–8, we detected a cluster of four genes, *snail*, *tinman*, *twist*, and *tkv *(*thickveins*) that share very similar patterns and eigen-profiles. They also have comparable patterns for several other stages. Interestingly, these genes are known determinants of mesoderm in Drosophila. Both *snail *and *twist *are activated by highest levels of the *dorsal *nuclear gradient in the ventral-most region of the early embryo (blastoderm stage 4–6). The gene *tinman *is activated in turn by *twist*, reaching a peak of expression in the invaginating mesoderm (gastrulation stage 7–8) and is a conserved key regulator of cardiac identity during mesodermal differentiation. Activity of *tkv *in the entire mesoderm induces ectopic *tinman *expression in the ventral mesoderm, and this results in the ectopic formation of heart precursors in a defined area of the ventrolateral mesoderm [22]. Thus, this cluster demonstrates that our method can detect transcriptional regulators and targets in a functional network of the early fly embryo.

So given such clusters of co-expressed genes, we then attempted to detect relevant sequence motifs using the cluster and eight related fly species *D. melanogaster*, *D. simulans*, *D. yakuba*, *D. erecta*, and *D. ananassae*, *D. pseudoobscura*, *D. virilis*, and *D. mojavensis*. To prove the principle we used several different sequence motif search tools – PhyloCon [23], PhyME [24], MEME [25] and MAST [26] – to find conserved motifs in the complete upstream regions in *D. melanogaster *of a cluster of co-expressed genes, *S*_*Q*_, and in the syntenically corresponding upstream regions in the other seven genomes, giving us 8*C *regions in which to find a common motif given the cluster contains *C *genes. Each motif was then used to scan the entire *D. melanogaster *genome to retrieve the set of genes, *S*_*R*_, for which an abundance of this motif is detected in their upstream regions. The expression patterns of genes in *S*_*R *_were then compared against those of genes in *S*_*Q*_. As an example, for the cluster *S*_*Q *_in Figure 2, three predicted motifs are shown. For each, we give the gene expression patterns and BDGP gene ontology annotations of two or three genes in *S*_*R*_*-S*_*Q*_. The gene expression patterns of the retrieved genes are visibly similar to those of the query genes over all developmental stages. This is also consistent with the genes sharing a lot of common BDGP annotations, such as TMA (trunk mesoderm anlage), VEA (ventral ectoderm anlage) for stage 7–8, and VNCP (ventral nerve cord primordium) for stage 9–10, suggesting that the detected motifs may be meaningful. This example of motif prediction-verification demonstrates the strength of the co-expressed/co-regulated gene detection based on our image clustering approach.

**Figure 3:**
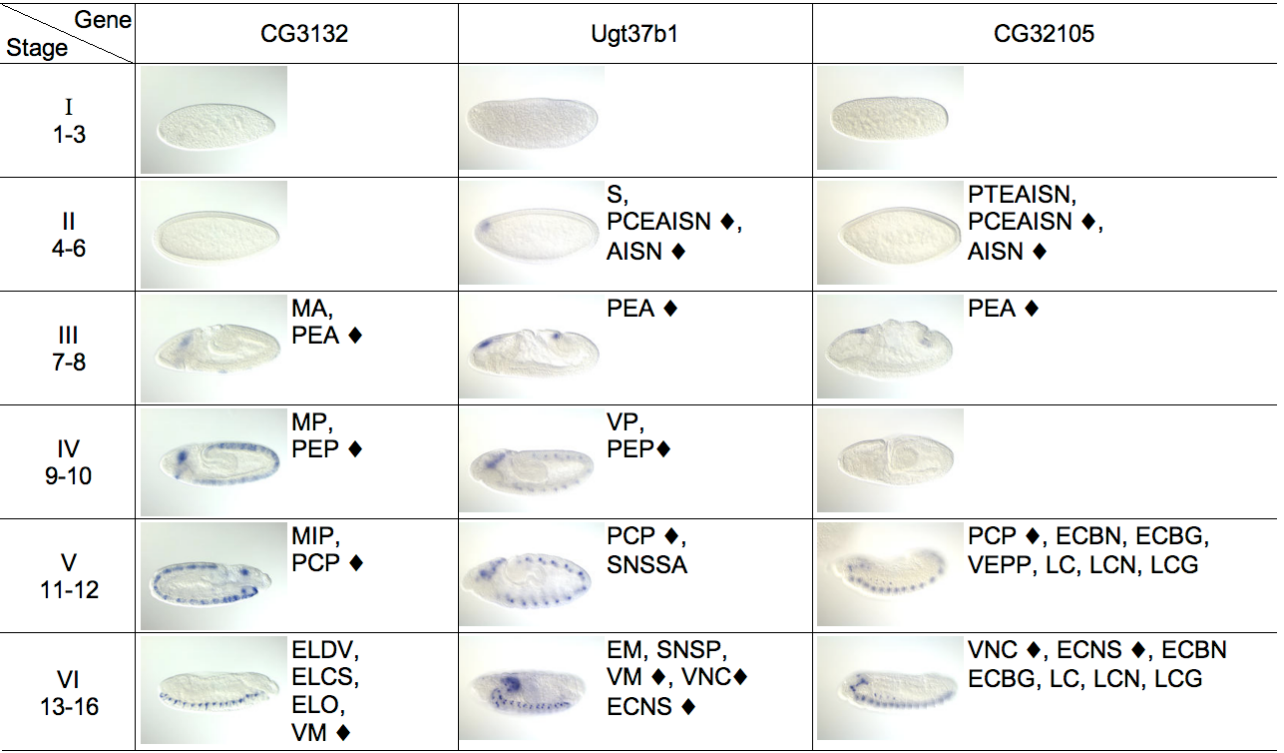
**A predicted group of three co-expressed genes CG3132, Ugt37b1, and CG32105, which have similar spatio-temporal patterns.** For each gene at each phase, one representative embryo image is shown, followed by the manual annotations extracted from BDGP. A "◆" is used to mark the common annotations (see Appendix A for abbreviations.)

Figure 3 shows another example, where the respective expression images of the three genes, CG3132, Ugt37b1, and CG32105, were always grouped into the same cluster for all six developmental stage-ranges. This indicates that they share common spatial expression patterns during the entire course of embryogenesis. We compared our predictions with the BDGP annotations listed besides the images. For instance, in stages 7–8, all three genes share the ontology term "PEA (procephalic ectoderm anlage)" indicated by a "◆"; in stages 11–12, they all share the term "PCP (protocerebrum primordium)"; and in stages 13–16, they seem to all be expressed in a subset of the nervous system, although gene CG3132 was not so annotated by the human curator. Thus the result is consistent with manual ontology annotations and demonstrates that our method can find co-expressed genes with similar patterns over the entire course of embryogenesis.

### Automatic annotation of gene expression patterns

Assigning descriptive terms to image patterns is very useful for both the qualitative and quantitative comparison of gene expression patterns, as well as their efficient organization, storage, and retrieval. Traditionally this task has been accomplished manually by expert annotators. In recent years, much of this kind of knowledge has been organized into controlled vocabularies or ontologies, so that it is possible to design automatic systems that assign such terms to gene expression patterns.

A gene expression pattern is often assigned several ontology terms corresponding to different local regions of the pattern covering a particular anatomically important area. As such a global basis representation, such as an eigen-profile, is not appropriate for this task. So we turned to the multi-resolution wavelet-profile of a gene expression pattern, and for each ontology term, we selected the top 20 MRMR wavelet-features discriminating that term in a training set [10]. A distinct classifier, based on a distinct MRMR-selected subset of wavelet features, was trained independently, and then all classifiers were run in parallel to deliver a set of terms for each image.

One difficulty we encountered is that the ontology annotation distribution is often skewed: the percentage of image samples that have a specific annotation is usually far less than that of the images without this annotation. Thus it is important to choose a pattern classifier that is robust to such a skew. We compared several classifiers, including Linear Discriminant Analysis (LDA) [20,27], Support Vector Machine (SVM) [28,20], and Quadratic Discriminant Analysis (QDA) [20,27] using the same set of 456 genes in the clustering analysis of the previous subsection. Table 1 shows the recognition rates of the LDA classifier for 5 gene ontology annotation terms used in stages 11–12. For each annotation, we partitioned the images of genes into a training set and a testing set by randomly picking 100 samples for testing and using the remaining samples for training. In these data sets, 15% or less of the samples have the annotated gene expression patterns. We generated 10 sets using random partition and show in Table 1 the average recognition rate on these random testing sets. We also computed the mean rate for the 5 annotations. Since the annotation distributions are unbalanced, we computed both the recognition rate on the small class, R_S_, and the overall recognition rate, R_O_. Table 1 shows that for LDA the R_S _values are close to the respective R_O _values, e.g. for HPP the values are 86% and 83%, respectively. These comparable recognition accuracies indicate that LDA is stable for skewed data distribution. In contrast, although SVM has higher overall recognition accuracy than LDA consistently, and QDA has the similar overall accuracy as LDA, their recognition rates on the small class are as low as 20%~67%, much lower than the respective overall accuracies. This unbalanced performance is undesirable for the purpose of automatic annotation.

**Table 1:** Recognition rate (%) of the LDA, SVM and QDA classifiers.

Classifier	*R*_*S *_(Recognition rate on the small class, i.e., the set of images that are annotated)						*R*_*O *_(Overall Recognition Rate)					
	
	HPP	PMP	AMP	PP	DEDP	Mean	HPP	PMP	AMP	PP	DEDP	Mean
LDA	86	82	82	87	84	84.2	83	80	84	86	88	84.2
SVM	27	19	25	67	57	39.0	91	89	91	97	95	92.6
QDA	20	23	35	38	23	27.8	85	82	83	87	86	84.6

Tests were conducted on images in stage 11–12. Recognition rates were calculated by taking the manual annotations as the ground truth.

Another difficulty we encountered was that of assigning multiple annotations to an image pattern, without knowledge of how many terms actually apply. To this end, each LDA classifier produces a probabilistic confidence score [10] estimating the likelihood that the annotation applies to a given image. The user can then ask the system to supply all annotations that have a likelihood above a given level, or ask for the *k*-best annotations in ranked order. Moreover, since each classifier is completely independent of every other, one can select a particular subset of classifiers, say corresponding to a general anatomical category such as nervous system, to run on a given set of images.

**Figure 4:**
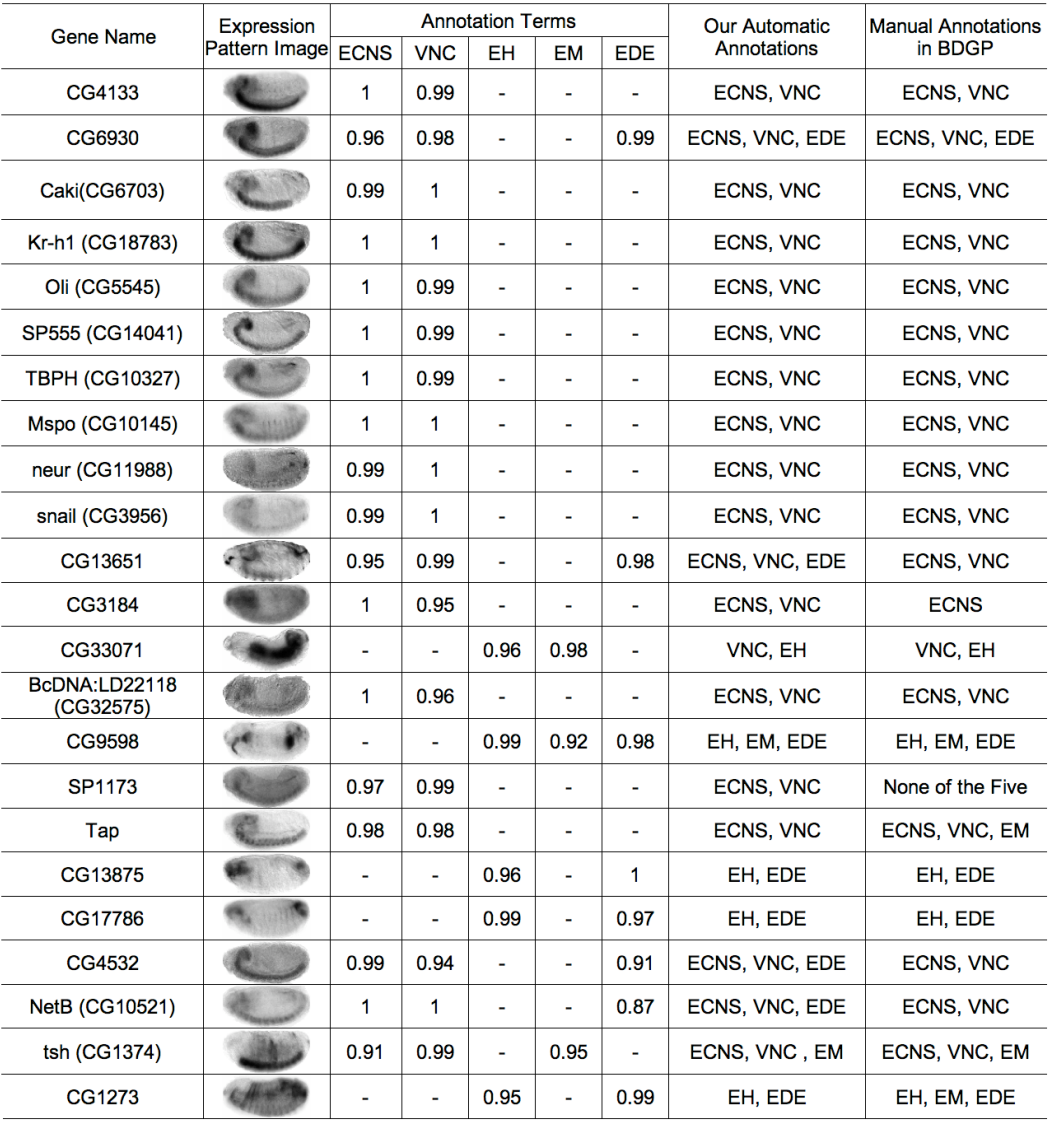
Predicted annotations for images at stage 13 – 16.

For the 456 genes used for the clustering study, we built a system to automatically annotate 70 of the gene ontology terms that have been used in the BDGP. The 70 terms corresponding to those that were manually associated with at least 6 images in a given stage range. For each of these a classifier was trained using the top 20 wavelet-embryo features selected by MRMR [11]. For testing purposes, the experiments were performed using leave-one-out cross-validation. The predicted annotations for a target gene were compared against the BDGP manual annotations. The URL [29] gives the complete set of annotations. Figure 4 shows an example of the annotation predictions, along with the estimated probabilities given by our annotation system. Entries with an estimated probability of lower than 0.6 are marked with "-", indicating that our system does not have high confidence in the prediction. Most of the annotations are consistent with expert's manual choices. For example, even when the image pattern for gene *snail *is blurred, our system can still predict correctly the two annotations "ECNS (embryonic central nervous system)" and "VNC (ventral nerve cord)". These examples demonstrate that our method can be applied to the automatic annotation of gene expression patterns.

We also made use of MRMR-selected wavelet-features to predict the developmental stage of an image, in addition to recognizing the anatomical regions in which a gene is expressed. As shown in the class prediction matrix of Table 2, over all 2,452 images for our test set of 456 genes, there were only 11 prediction errors in a 10-fold cross-validation scheme, for an overall automatic staging accuracy of 99.55%. This further demonstrates the effectiveness of the wavelet-feature representation.

**Table 2:** Prediction (image numbers) of the developmental stage of images.

Actual stage range/Predicted stage range	1–3	4–6	7–8	9–10	11–12	13–16
1–3	447	3	0	0	0	0
4–6	0	446	0	0	0	0
7–8	1	0	379	0	0	0
9–10	0	0	0	372	1	0
11–12	1	1	0	0	435	0
13–16	2	0	0	0	2	362

## Discussion

This study has focused on the 2D *in situ *hybridization patterns within images generated by the BDGP. The results in this paper, and those of our earlier work [8]-[10], show that our image analysis methods can be effective in automatically analyzing a large-scale ISH dataset, e.g. the 10's of thousands of embryonic expression patterns available for the 5,000 *D. melanogaster *genes processed thus far by the BDGP.

In Figure 2 we showed that motifs can be detected by combining image clustering and comparative sequence analysis. The results can be further improved by building a more sophisticated model that incorporates information from (a) the images, (b) the known evolutionary distances between the genomes, and (c) the enrichment of the potential motifs. The ultimate verification of these predicted motifs will rely on experimental examination of the respective enhancers regions.

In regard to the automatic annotation of gene expression patterns, we also collected statistics of the use of the ontology annotation terms (results not shown). The percentage of genes corresponding a common annotation term ranges from less than 1% to about 20%. Given this small fraction, it is unlikely that our image-based gene clustering results in Figures 2 and 3, as well as the respective consistent BDGP annotations, could have been obtained simply because these patterns and annotations were ubiquitous. A more quantitative analysis of these results, as well as sequence motifs will be given in a separate paper.

Our image analysis methods can be applied to 3D gene expression patterns or other types of aligned image patterns in different contexts. For example, in [30] we defined similarity scores between gene expression patterns of 3D multiplex stained fly embryos and developed a minimum spanning tree based method to temporally sort the images, giving us a reconstruction of the developmental dynamics of the expression patterns. We can further use the methods introduced in this paper to analyze these temporally "sorted" 3D embryo patterns.

The key issues discussed in this paper, namely how to define and compare gene expression patterns, and how to cluster and annotate them, are general problems for many datasets other than just fly embryonic gene expression patterns. For example, for recently published Allen Brain Atlas of mouse brain gene expression patterns [31,32], *in situ *hybridization brain images of 20,000 genes were aligned to a standard reference atlas. The effective clustering and recognition of these gene expression patterns, based on various image features (e.g. [33]), could contribute significantly to inferring a whole-brain, whole-genome correlation graph of gene expression.

## Conclusion

We have developed a set of automatic image analysis methods for *in situ *fly gene expression patterns. We have successfully extracted useful local and global image features, and used these to automatically cluster and annotate gene expression patterns with high reliability. Our techniques provide useful tools for the large-scale computational screening of fly embryonic gene expression patterns, as well as the aligned image patterns for similar problems in other model systems.

## Data

For the BDGP database, 2D embryonic images of gene expression patterns were acquired using a digital camera. In a typical image, a single embryo resides in the central part of the image and presents a lateral view. Only lateral views were used, but the embryo can otherwise have an arbitrary orientation. As the embryonic region has much richer texture information than the image background, the embryo can be segmented by thresholding the local variance of a small region (e.g. 3 × 3 pixels) around each pixel. The pixel is binarized to "foreground" if the variance is larger than a predefined threshold (e.g. 2), otherwise to "background". Binarization classifies most embryo pixels as "foreground" and most background pixels as "background", thus producing a mask image that essentially captures the embryonic region. For a segmented embryo, we computed the principal direction along which the variation of all embryonic pixel-coordinates is the greatest and considered this the anterior-posterior axis of the embryo. We then rotated the image to make this axis horizontal. Finally, the embryonic region was cropped and its size was standardized to 400 pixels wide and 200 pixels high.

We processed about 30,000 *in situ *gene expression images in the BDGP database for 1,700 fly genes. We found that about 67% of the images have only one embryo region in the center, and can be easily segmented based on thresholding the pixel variance. These 20,000 extracted image patterns were automatically rotated so that their longest axes are horizontal. We developed a web-based image pattern browser at [34], which can compare the extracted embryonic regions of multiple genes simultaneously, an important function currently missing in the BDGP database. This browser also provides links to the gene expression patterns, microarray expression data, and gene ontology annotations within the BDGP database and to other gene information in FlyBase [35].

In analyzing the data, we further ignored all images that were not of a lateral view of the embryo. While most images are lateral views, a significant fraction is taken from difference vantage points, such as along the dorsal/ventral axis or some tilted angle. From these viewpoints it is especially difficult to understand the 3D pattern as the embryo is clear and one is essentially seeing the 2D projection of the stain along the viewing axis. It remains an open problem how to effectively use such additional data.

For the experimental results reported, we focused on a set of 456 genes. We separated the lateral and dorsal views manually and also adjusted the orientations of these images to assure these images are compared in the correct way, i.e. anterior is at the left and dorsal is up. If in a particular stage-range a gene has multiple images, our computer program merged these images and used their mean-image as the "representative" for this gene. In this way, the image clustering and annotation algorithms would not be biased by the image-numbers of genes. Due to the great variation of the quality of the BDGP 2D image patterns, these processing steps were necessary to produce meaningful results.

## Appendix A

Abbreviations of the anatomical annotations used throughout the paper:

AM amnioserosa

AAISN amnioserosa anlage in statu nascendi

AISN anlage in statu nascendi

AEA anterior endoderm anlage

AEAISN anterior endoderm anlage in statu nascendi

AEP anterior endoderm primordium

AMP anterior midgut primordium

CMP cardiac mesoderm primordium

CB cellular blastoderm

CLP clypeo-labral primordium

DEA dorsal ectoderm anlage

DEAISN dorsal ectoderm anlage in statu nascendi

DECP dorsal ectoderm primordium

DEDP dorsal epidermis primordium

DPMP dorsal pharyngeal muscle primordium

EAISN endoderm anlage in statu nascendi

ECBG embryonic central brain glia

ECBN embryonic central brain neuron

ECNS embryonic central nervous system

EDE embryonic dorsal epidermis

EH embryonic hindgut

ELDV embryonic/larval dorsal vessel

ELCS embryonic/larval circulatory system

ELO embryonic/larval oenocyte

EM embryonic midgut

EOLP embryonic optic lobe primordium

EFP external foregut primordium

FA foregut anlage

FAISN foregut anlage in statu nascendi

FP foregut primordium

GC germ cell

HMPP head mesoderm P2 primordium

HMA head mesoderm anlage

HA hindgut anlage

HPP hindgut proper primordium

IHP inclusive hindgut primordium

LC lateral cord

LCG lateral cord glia.

LCN lateral cord neuron

LVMP longitudinal visceral mesoderm primordium

MA mesectoderm anlage

MEP mesectoderm primordium

MIP midline primordium

MAISN mesoderm anlage in statu nascendi

MLP midline primordium

MP mesectoderm primordium

NOVNS neuroblasts of ventral nervous system

OSA oenocyte specific anlage

PC pole cell

PTEA posterior endoderm anlage

PTEP posterior endoderm primordium

PMP posterior midgut primordium

PCEA procephalic ectoderm anlage

PCEAISN procephalic ectoderm anlage in statu nascendi

PCEP procephalic ectoderm primordium

PCN procephalic neuroblasts

PEA procephalic ectoderm anlage

PEP procephalic ectoderm primordium

PCP protocerebrum primordium

PMP posterior midgut primordium

PP proventriculus primordium

PTEAISN posterior endoderm anlage in statu nascendi

SDP salivary duct primordium

SGBSA salivary gland body specific anlage

SGDSA salivary gland duct specific anlage

SGBP salivary gland body primordium

SNSSA sensory nervous system specific anlage

SMP somatic muscle primordium

S subset

TP tracheal primordium

SNSP sensory nervous system primordium

SNSSA sensory nervous system specific anlage

TMA trunk mesoderm anlage

TMAISN trunk mesoderm anlage in statu nascendi

TMP trunk mesoderm primordium

VEA ventral ectoderm anlage

VECP ventral ectoderm primordium

VEPP ventral epidermis primordium

VNCP ventral nerve cord primordium

VNA ventral neuroderm anlage

VSCSA ventral sensory complex specific anlage

VM ventral midline

VMP visceral muscle primordium

VNC ventral nerve cord

VP visual primordium

## Competing interests

The authors declare that they have no competing interests.
